# Supplementary material for: Chondrule-like objects and Ca-Al-rich inclusions in Ryugu may potentially be the oldest Solar System materials
Source: Nat Commun. 2023 Feb 16;14:532. doi: 10.1038/s41467-023-36268-8 (PMC9935534; doi:10.1038/s41467-023-36268-8)
Supplement: Supplementary file 2 — Supplementary information [file 41467_2023_36268_MOESM2_ESM.pdf]

Supplementary Table 1: Major element compositions of chondrule-like objects and CAIs in the Ryugu samples measured with FE-EPMA[a] and TEM[b]

Sample name	Analysis #[c]	Target	SiO_2	TiO_2	Al_2O_3	Cr_2O_3	FeO	MnO	MgO	CaO	Na_2O	NiO	K_2O	SO_3	P_2O_5	Total	Mg#
	1499	Ol	42.390	0.022	0.052	0.592	1.560	0.413	56.240	0.182	0.039	0.090	n.m.	n.m.	n.m.	101.58	98.5
C0002-P5-C1-Chd	1500	Ol	42.900	0.012	0.011	0.682	1.450	1.510	55.680	0.164	0.051	0.091	n.m.	n.m.	n.m.	102.55	98.6
	1501	Ol	42.400	0.038	0.042	0.549	1.350	0.320	55.790	0.191	0.051	0.076	n.m.	n.m.	n.m.	100.80	98.7
C0002-P5-C2-Chd	1476	Ol	42.660	0.000	0.020	0.276	1.169	0.472	58.020	0.020	0.014	0.132	n.m.	n.m.	n.m.	102.79	98.9
	1477	Ol	41.090	0.004	0.012	0.301	1.290	0.484	56.110	0.018	0.001	0.085	n.m.	n.m.	n.m.	99.39	98.7
	2496	Ol	46.990	0.020	0.094	0.328	1.155	0.464	46.590	3.030	0.021	0.124	n.m.	n.m.	n.m.	98.82	98.6
	TEM-1	Ol	41.636	0.170	0.000	0.448	0.424	1.005	56.129	0.132	0.000	0.000	0.056	n.m.	n.m.	100.00	99.6
	TEM-2	Ol	42.171	0.000	0.000	0.535	0.253	0.916	55.994	0.131	0.000	0.000	0.000	n.m.	n.m.	100.00	99.7
	TEM-3	Ol	42.311	0.000	0.000	0.181	0.000	0.761	56.748	0.000	0.000	0.000	0.000	n.m.	n.m.	100.00	100.0
	TEM-4	Ol	42.487	0.180	0.000	0.269	0.509	0.502	55.941	0.066	0.000	0.000	0.056	n.m.	n.m.	100.00	99.5
C0040-02-Chd	TEM-5	Ol	41.975	0.085	0.000	0.361	0.341	0.843	56.394	0.000	0.000	0.000	0.000	n.m.	n.m.	100.00	99.7
	TEM-6	Di	55.217	0.230	0.094	0.263	0.026	0.207	20.992	22.919	0.000	0.000	0.060	n.m.	n.m.	100.00	99.9
	TEM-7	Di	55.260	0.000	0.397	0.000	0.270	0.013	20.942	23.283	0.000	0.000	0.000	n.m.	n.m.	100.16	99.3
	TEM-8	Di	56.522	0.030	0.019	0.190	0.180	0.000	20.892	22.163	0.000	0.000	0.000	n.m.	n.m.	100.00	99.5
	TEM-9	Di	56.108	0.000	0.298	0.355	0.084	0.166	20.994	21.994	0.000	0.000	0.000	n.m.	n.m.	100.00	99.8
	TEM-10	Di	55.102	0.370	0.178	0.000	0.167	0.082	20.646	23.436	0.000	0.000	0.055	n.m.	n.m.	100.00	99.6
	2482	Sp	0.091	0.931	73.670	0.093	0.816	0.000	24.340	1.249	0.000	0.058	n.m.	n.m.	n.m.	101.25	
	2483	Sp	0.058	0.768	73.620	0.110	0.853	0.000	24.860	1.149	0.004	0.067	n.m.	n.m.	n.m.	101.49	
	2484	Hib	0.162	5.500	80.230	0.067	0.855	0.008	7.100	6.760	0.001	0.000	n.m.	n.m.	n.m.	100.68	
	2485	Hib	0.233	5.050	79.280	0.109	0.761	0.019	9.310	6.050	0.008	0.071	n.m.	n.m.	n.m.	100.89	
	2486	Sp	0.348	0.035	71.540	0.141	0.892	0.000	27.720	0.014	0.002	0.047	n.m.	n.m.	n.m.	100.74	
C0040-02-CAI	2487	Sp	1.288	0.232	67.800	0.121	1.029	0.000	27.550	0.110	0.018	0.031	n.m.	n.m.	n.m.	98.18	
	2811	Phyl	19.733	0.088	16.106	1.003	12.082	0.207	28.914	0.111	0.362	0.286	0.033	5.627	0.289	84.84	
	2812	Phyl	18.538	0.083	17.751	0.329	6.685	0.311	28.183	0.071	0.101	0.053	0.002	5.426	0.418	77.95	
	2813	Phyl	19.270	0.060	17.221	0.907	10.032	0.243	30.197	0.188	0.319	0.294	0.012	4.976	0.217	83.94	
	2814	Phyl	21.556	0.131	15.134	0.479	8.752	0.291	28.347	0.075	0.147	0.149	0.037	5.309	0.354	80.76	
	2815	Phyl	21.545	0.085	14.083	1.619	13.780	0.203	24.837	0.231	0.671	0.577	0.037	4.107	0.112	81.89	
	5845	Sp	0.210	0.172	70.420	0.154	0.712	0.000	28.440	0.020	0.000	0.073	n.m.	n.m.	n.m.	100.20	
	5846	Sp	0.500	0.191	70.470	0.149	0.676	0.002	28.430	0.016	0.002	0.023	n.m.	n.m.	n.m.	100.46	
	5847	Sp	0.758	0.235	69.610	0.151	0.697	0.009	27.740	0.008	0.009	0.000	n.m.	n.m.	n.m.	99.22	
	5848	Sp	0.525	0.175	71.340	0.145	0.707	0.008	28.700	0.005	0.007	0.050	n.m.	n.m.	n.m.	101.66	
C0076-10-CAI	5995	Phyl	35.573	0.097	21.736	0.435	5.463	0.070	27.765	0.263	0.109	0.381	0.001	0.898	0.075	92.87	
	5996	Phyl	44.318	0.104	7.891	0.474	5.654	0.148	22.368	0.127	0.232	0.543	0.030	1.027	0.063	82.98	
	5997	Phyl	34.901	0.093	3.631	0.440	8.596	0.230	19.874	0.588	0.149	0.572	0.021	2.163	0.109	71.37	
	5998	Phyl	40.911	0.073	5.108	0.444	4.975	0.109	17.203	0.115	0.196	0.367	0.021	0.842	0.089	70.45	
	5999	Phyl	34.076	0.091	3.239	0.436	8.726	0.145	19.287	0.113	0.060	0.617	0.000	2.575	0.049	69.41	
	6000	Phyl	37.744	0.099	3.173	0.566	6.476	0.181	21.578	0.296	0.120	0.499	0.044	1.418	0.103	72.30	

[a] Detection limits of individual elements are shown in Nakamura et al.[1].

[b] Total wt% is normalized to 100, and TEM analysis spots are not shown in any figure.

[c] Analysis spots are shown in supplementary figures.

Supplementary Table 2: Raw SIMS measured oxygen-isotope data of chondrule-like objects and CAIs in the Ryugu samples

Analysis_#	Sample	$\delta^{18}O$ ‰ RAW	2SE	$\delta^{17}O$ ‰ RAW	2SE	$\Delta^{17}O$ ‰ RAW	2SE	^{16}O (Mcps)	OH correction (‰)[a]	IMF[b]	IMF	$\delta^{18}O$ ‰	Error (2SD)	$\delta^{17}O$ ‰	Error (2SD)	$\Delta^{17}O$ ‰	Error (2SD)
20220424_52	3Hole_Disk_SCOl (San Carlos Olivine)	0.69	2.68	0.75	4.05	0.39	4.28	0.32	0.42								
20220424_53	3Hole_Disk_SCOl	0.26	2.02	-0.43	3.47	-0.56	3.63	0.32	0.45								
20220424_54	3Hole_Disk_SCOl	1.09	2.69	8.90	4.90	8.33	5.10	0.32	0.42								
	**Average Standard & 2SD for C0002-P5-C2-Chd & C1-Chd/52-54, 61-63**	**0.78**	**1.96**	**1.64**	**7.91**	**1.24**	**8.47**				**-4.51**						
20220424_57	3Hole_C0002-P5_C2_Chd spot1_Ol	-44.96	3.98	-45.22	5.94	-21.84	6.29	0.29	0.34	0.22	-4.30	**-39.81**	**1.96**	**-43.63**	**7.91**	**-22.93**	**8.47**
20220424_58	3Hole_C0002-P5_C2_Chd spot2_Ol	-52.05	1.73	-48.85	5.97	-21.78	6.04	0.28	0.34	0.22	-4.30	**-47.49**	**1.96**	**-47.82**	**7.91**	**-23.13**	**8.47**
20220424_59	3Hole_C0002-P5_C1_Chd spot1_Ol	-1.52	3.55	-2.69	6.33	-1.90	6.60	0.21	0.53	0.22	-4.30	**2.56**	**1.96**	**-2.46**	**7.91**	**-3.79**	**8.47**
20220424_60	3Hole_C0002-P5_C1_Chd spot2_Ol	-5.99	3.16	-4.77	6.23	-1.65	6.44	0.20	0.30	0.22	-4.30	**-1.35**	**1.96**	**-3.73**	**7.91**	**-3.03**	**8.47**
20220424_61	3Hole_Disk_right_SCOl	-0.52	2.21	4.69	4.60	4.96	4.74	0.33	0.37								
20220424_62	3Hole_Disk_right_SCOl	1.20	2.36	1.24	6.08	0.62	6.21	0.32	0.37								
20220424_63	3Hole_Disk_right_SCOl	1.90	2.04	-2.96	5.00	-3.94	5.11	0.32	0.38								
20220424_95	7 hole_Disk, SC-Ol	4.18	2.52	3.50	5.86	1.33	6.00	0.32	0.16								
20220424_96	7 hole_Disk, SC-Ol	4.58	2.12	6.33	3.81	3.94	3.96	0.33	0.15								
20220424_97	7 hole_Disk, SC-Ol	1.29	2.40	3.79	5.76	3.12	5.89	0.33	0.15								
	**Average Standard & 2SD for C0040-02-CAI/95-97, 102-104 (N=6)**	**1.81**	**2.43**	**2.09**	**4.81**	**1.15**	**4.05**				**-3.49**						
20220424_98	7 hole_C0040-02-CAI spot1_Hib	-46.46	2.61	-49.21	6.43	-25.04	6.57	0.23	0.44	-3.04	-6.52	**-39.11**	**2.43**	**-46.47**	**4.81**	**-26.13**	**4.05**
20220424_99	7 hole_C0040-02-CAI spot2_Sp	-51.45	2.80	-46.45	6.94	-19.69	7.09	0.23	0.24	-3.33	-6.81	**-43.14**	**2.43**	**-42.65**	**4.81**	**-20.22**	**4.05**
20220424_10[illegible]	7 hole_C0040-02-CAI spot3_Sp	-47.61	3.12	-44.67	7.62	-19.91	7.79	0.22	0.19	-3.33	-6.81	**-42.49**	**2.43**	**-44.01**	**4.81**	**-21.92**	**4.05**
20220424_10[illegible]	7 hole_C0040-02-CAI spot4_Sp	-50.26	3.31	-46.74	4.32	-20.60	4.65	0.23	0.32	-3.33	-6.81	**-43.09**	**2.43**	**-44.16**	**4.81**	**-21.75**	**4.05**
20220424_10[illegible]	7 hole_Disk, SC-Ol	1.57	2.98	1.07	5.49	0.25	5.70	0.32	0.18								
20220424_10[illegible]	7 hole_Disk, SC-Ol	0.63	1.85	-1.48	4.32	-1.81	4.43	0.34	0.16								
20220424_10[illegible]	7 hole_Disk, SC-Ol	0.98	2.02	2.67	4.06	2.16	4.20	0.33	0.15								
	**Average Standard & 2SD for C0040-02-Chd & C0076-10-CAI/102-104,**	**1.33**	**1.33**	**2.20**	**5.39**	**1.51**	**5.15**				**-3.97**						
20220424_10[illegible]	7 hole_C0040-02-Chd spot1_Ol	-48.28	2.52	-46.57	4.98	-21.46	5.15	0.29	0.11	0.22	-3.75	**-44.39**	**1.33**	**-46.02**	**5.39**	**-22.94**	**5.15**
20220424_10[illegible]	7 hole_C0076-10-CAI spot1_Sp	-51.65	2.34	-48.95	7.65	-22.09	7.74	0.23	0.16	-3.33	-7.29	**-43.96**	**1.33**	**-46.27**	**5.39**	**-23.41**	**5.15**
20220424_10[illegible]	7 hole_C0076-10-CAI spot2_Sp	-47.04	3.37	-47.72	6.44	-23.26	6.68	0.24	0.17	-3.33	-7.29	**-40.26**	**1.33**	**-45.99**	**5.39**	**-25.06**	**5.15**
20220424_10[illegible]	7 hole_Disk, SC-Ol	1.37	1.88	6.62	5.22	5.91	5.31	0.35	0.14								
20220424_10[illegible]	7 hole_Disk, SC-Ol	1.84	3.17	2.18	6.19	1.22	6.40	0.34	0.13								
20220424_11[illegible]	7 hole_Disk, SC-Ol	2.29	2.27	3.75	4.45	2.56	4.61	0.35	0.13								

[a]OH correction (‰) = $^{16}O^{1}H^{-}$ intensity/[$^{17}O^{-}$ intensity ×($^{17}O^{-}$ tail/$^{17}O^{-}$ intensity)]×1000; $^{17}O^{-}$ tail/$^{17}O^{-}$ intensity = 2.1×10^{-5}.

[b]Correction for instrumental bias using standards that have similar chemical compositions to the target phases.

Supplementary Table 3: The instrumental bias relative to SCOl (San Carlos Olivine) among different standards.

Standard name	Mineral	Standard					SCOL (bracketing analyses)					Bias relative to		laser Fluorination	
		$\delta^{18}O \pm 2SD$ (‰)		Bias ± 2SE (‰)[a]		N	$\delta^{18}O \pm 2SD$ (‰)		Bias ± 2SE (‰)		N	SCOL ± 2SE (‰)[b]		$\delta^{18}O$ (‰)	Ref
HN-Ol	Fo100	4.85	2.31	-4.02	1.16	4	1.06	4.12	-4.24	1.46	8	0.22	1.86	8.90	[2]
Spinel#1	Spinel	10.87	2.72	-6.20	1.36	4	2.42	1.03	-2.88	0.37	8	-3.33	1.41	17.18	[3]
Madagascar hibonite	Hibonite	4.85	0.99	-5.91	0.49	4	2.42	1.03	-2.88	0.37	8	-3.04	0.61	10.83	[3]

[a]Instrumental bias in $\delta^{18}O$ from $\delta^{18}O$ values determinted by laser fluorination.

[b]Correction factors of instrumental bias caused by chemical compositions of the chondrule-like objects and CAIs (matrix effect).

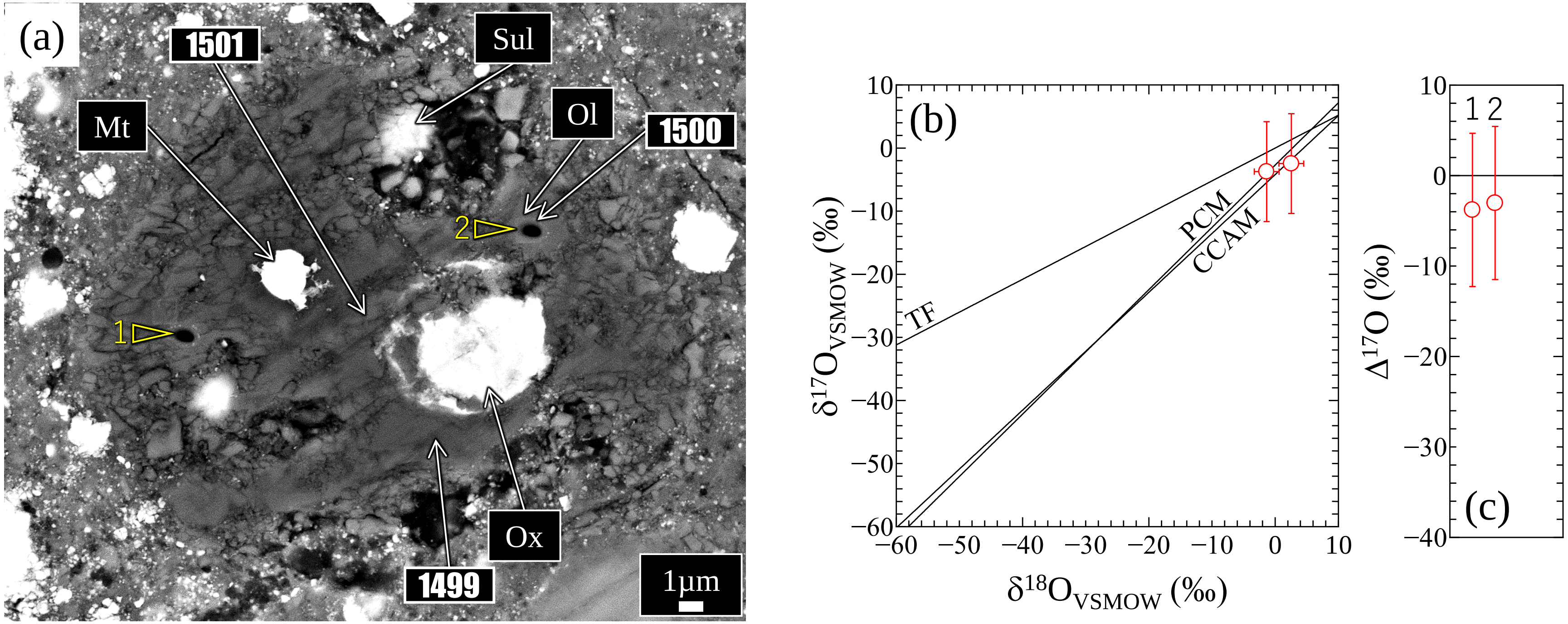

Supplementary Fig. 1: The BSE image of C0002-P5-C1-Chd (a), oxygen three-isotope ratios of C0002-P5-C1-Chd (b), and $\Delta^{17}O$ values of C0002-P5-C1-Chd (c). Four-digit numbers in panel a correspond to analysis numbers of FE-EPMA (Supplementary table 1). Abbreviations in panel a: Ol, olivine; Mt, Fe-Ni metal; Sul, Fe-sulfide; Ox, oxide. TF, PCM, and CCAM in panel b represent the Terrestrial Fractionation line, the Primitive Chondrule Mineral line[4], and the Carbonaceous Chondrite Anhydrous Mineral line[5]. Numbers in panel c correspond to analysis numbers of SIMS (Supplementary table 2).

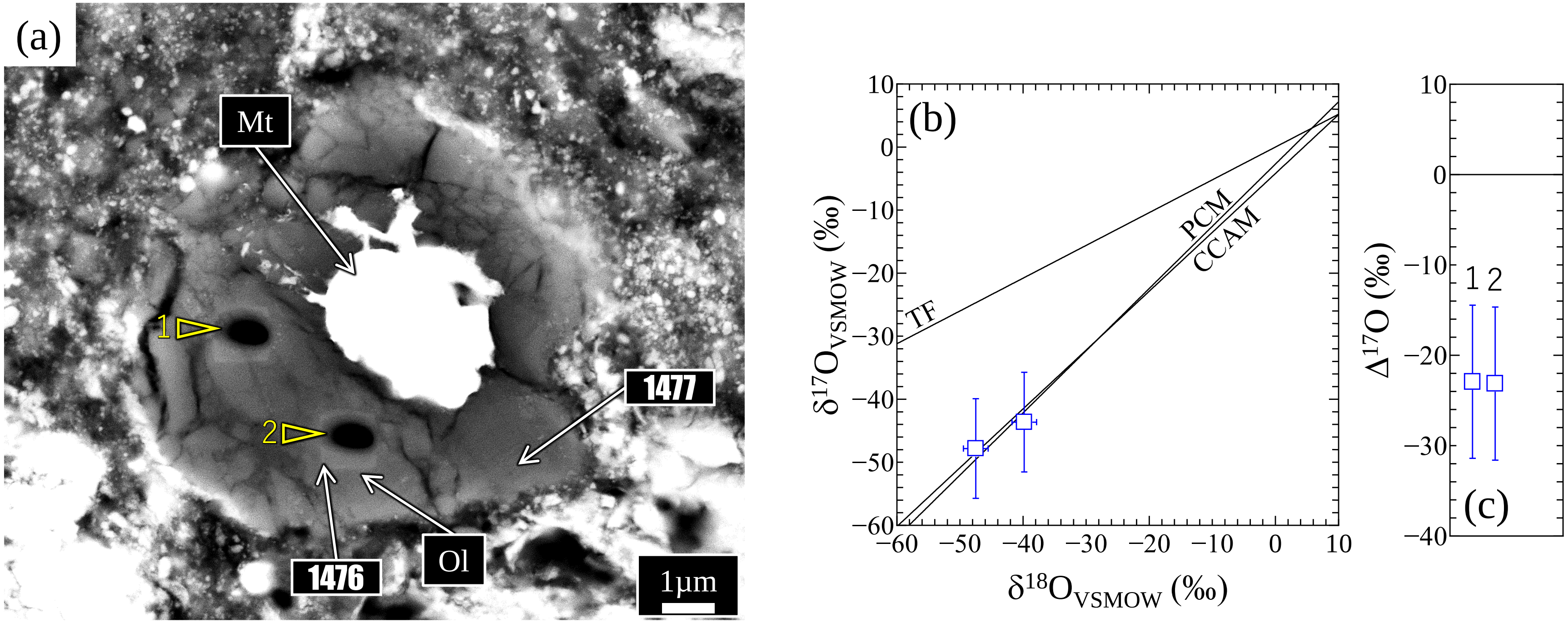

Supplementary Fig. 2: The BSE image of C0002-P5-C2-Chd (a), oxygen three-isotope ratios of C0002-P5-C2-Chd (b), and $\Delta^{17}O$ values of C0002-P5-C2-Chd (c). Four-digit numbers in panel a correspond to analysis numbers of FE-EPMA (Supplementary table 1). Abbreviations in panel a: Ol, olivine; Mt, Fe-Ni metal. TF, PCM, and CCAM in panel b represent the Terrestrial Fractionation line, the Primitive Chondrule Mineral line[4], and the Carbonaceous Chondrite Anhydrous Mineral line[5]. Numbers in panel c correspond to analysis numbers of SIMS (Supplementary table 2).

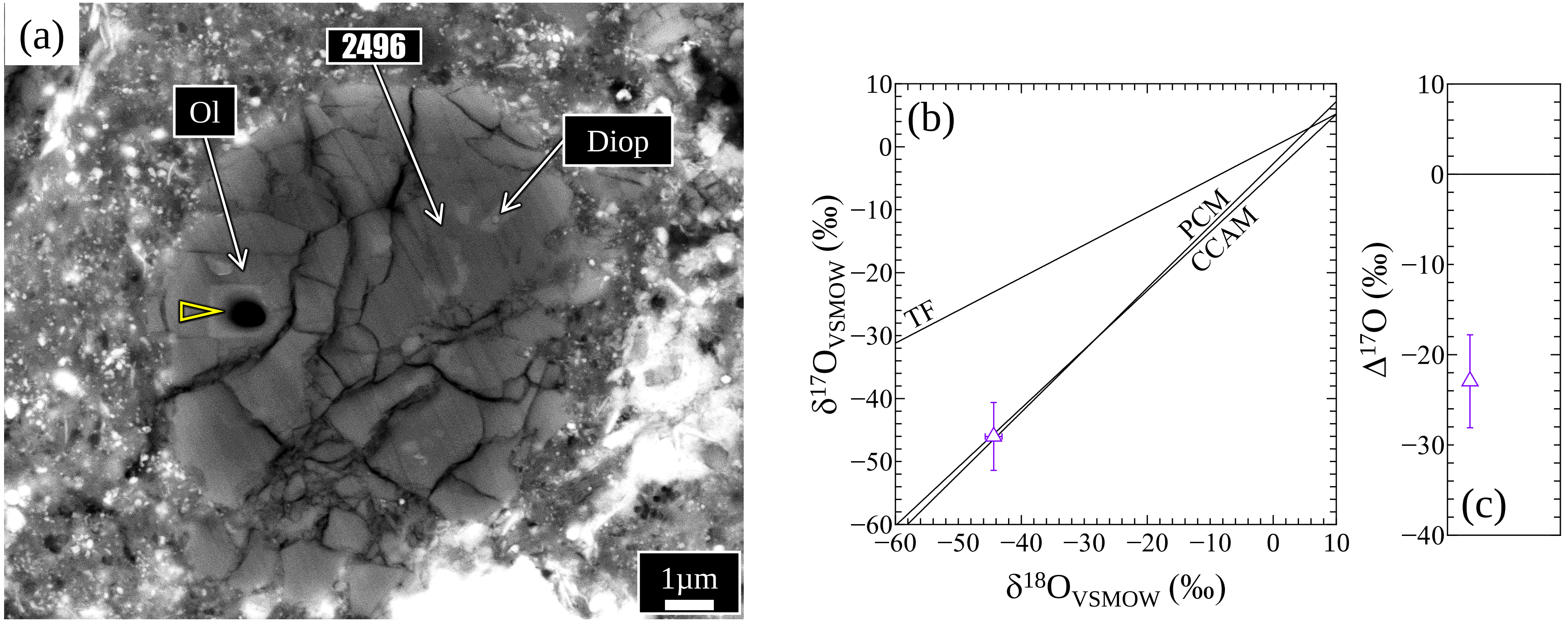

Supplementary Fig. 3: The BSE image of C0040-02-Chd (a), oxygen three-isotope ratios of C0040-02-Chd (b), and $\Delta^{17}O$ value of C0040-02-Chd (c). The four-digit number in panel a corresponds to the analysis number of FE-EPMA (Supplementary table 1). Abbreviations in panel a: Ol, olivine; Diop, diopside. TF, PCM, and CCAM in panel b represent the Terrestrial Fractionation line, the Primitive Chondrule Mineral line[4], and the Carbonaceous Chondrite Anhydrous Mineral line[5].

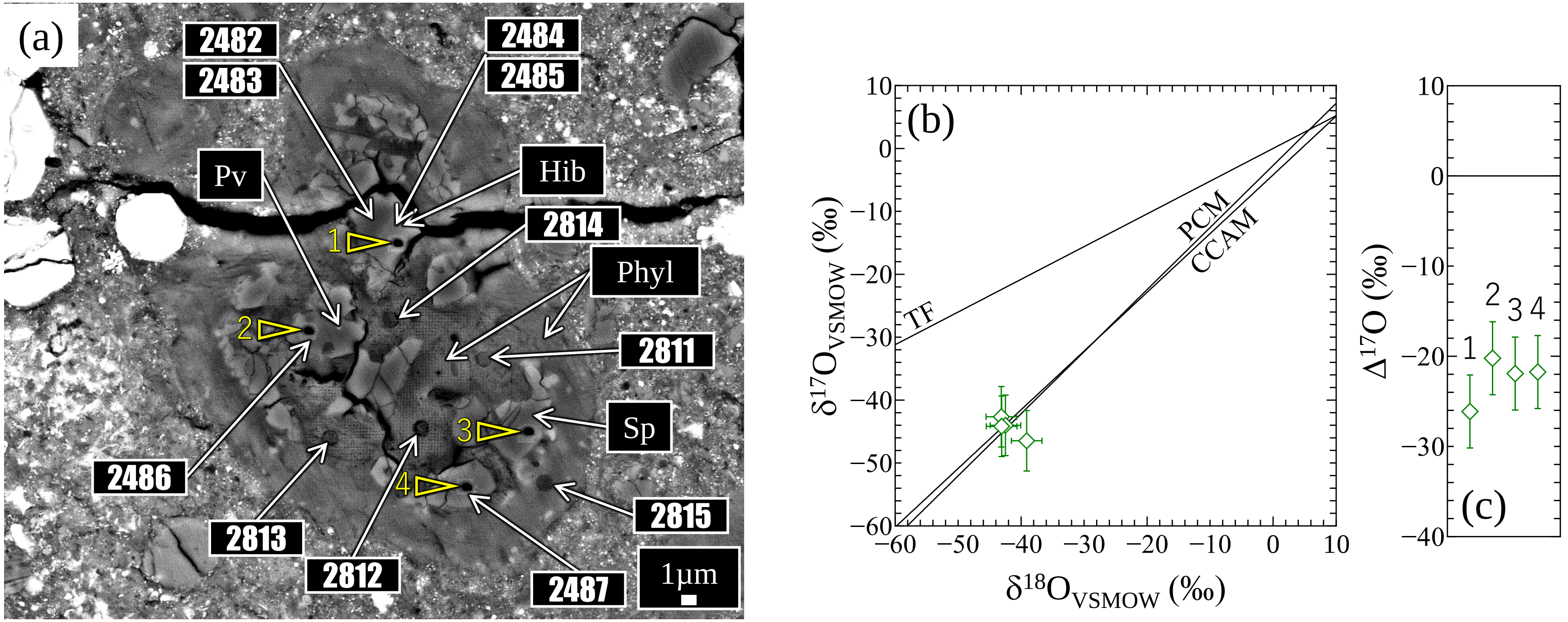

Supplementary Fig. 4: The BSE image of C0040-02-CAI (a), oxygen three-isotope ratios of C0040-02-CAI (b), and $\Delta^{17}O$ values of C0040-02-CAI (c). Four-digit numbers in panel a correspond to analysis numbers of FE-EPMA (Supplementary table 1). Abbreviations: Sp, spinel; Hib, hibonite; Pv, perovskite; Phyl, phyllosilicates. TF, PCM, and CCAM in panel b represent the Terrestrial Fractionation line, the Primitive Chondrule Mineral line[4], and the Carbonaceous Chondrite Anhydrous Mineral line[5]. Numbers in panel c correspond to analysis numbers of SIMS (Supplementary table 2).

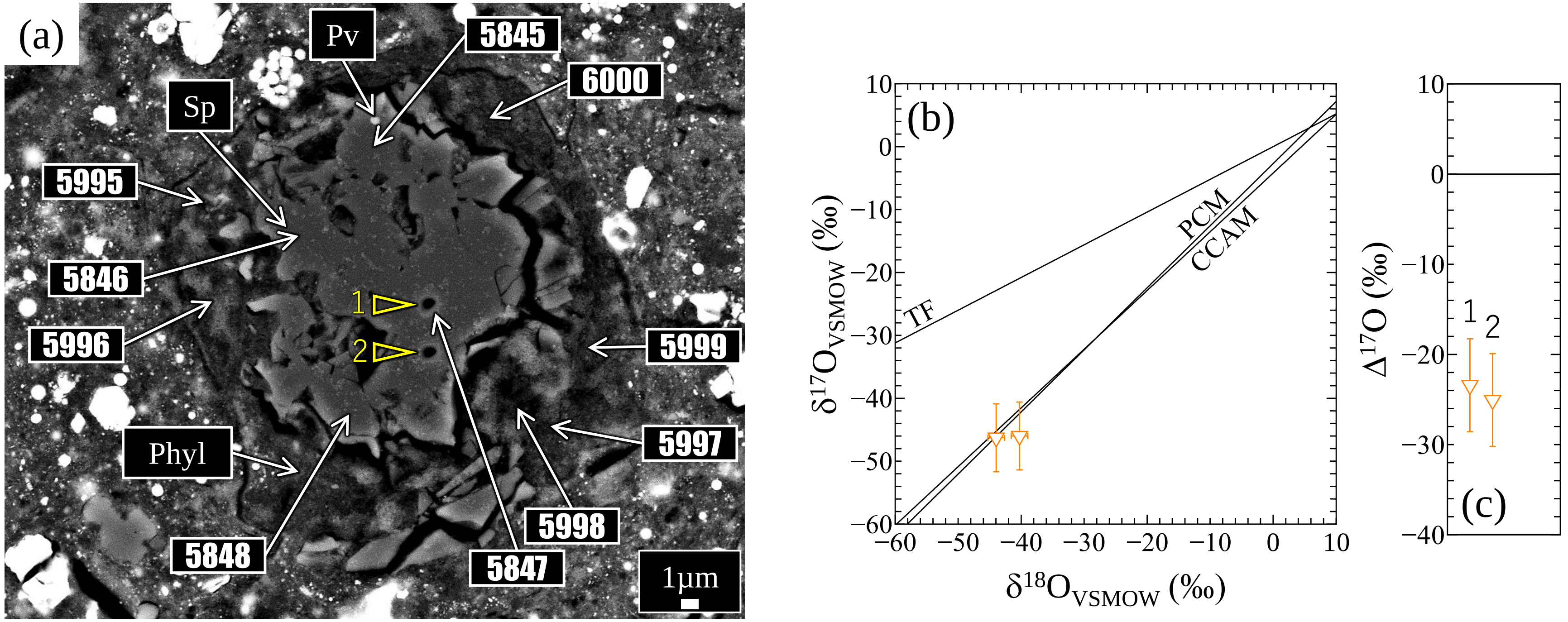

Supplementary Fig. 5: The BSE image of C0076-10-CAI (a), oxygen three-isotope ratios of C0076-10-CAI (b), and $\Delta^{17}O$ values of C0076-10-CAI (c). Four-digit numbers in panel a correspond to analysis numbers of FE-EPMA (Supplementary table 1). Abbreviations: Sp, spinel; Pv, perovskite; Phyl, phyllosilicates. TF, PCM, and CCAM in panel b represent the Terrestrial Fractionation line, the Primitive Chondrule Mineral line[4], and the Carbonaceous Chondrite Anhydrous Mineral line[5]. Numbers in panel c correspond to analysis numbers of SIMS (Supplementary table 2).

Supplementary reference

1. Nakamura, T. et al. Formation and evolution of Cb-type asteroid Ryugu: Direct evidence from returned samples. *Science* 10.1126/science.abn8671 (2022).
2. Kita, N. T. et al. High precision SIMS oxygen three isotope study of chondrules in LL3 chondrites: Role of ambient gas during chondrule formation. *Geochim. Cosmochim. Acta* **74**, 6610-6635 (2010).
3. Ushikubo, T., Tenner, T. J., Hiyagon, H., & Kita, N. T. A long duration of the ^{16}O-rich reservoir in the solar nebula, as recorded in fine-grained refractory inclusions from the least metamorphosed carbonaceous chondrites. *Geochim. Cosmochim. Acta* **201**, 103-122 (2017).
4. Ushikubo, T., Kimura, M., Kita, N. T., & Valley, J.W. Primordial oxygen isotope reservoirs of the solar nebula recorded in chondrules in Acfer 094 carbonaceous chondrite. *Geochim. Cosmochim. Acta* **90**, 242–264 (2012).
5. Clayton, R. N., Onuma, N., Grossman, L. & Mayeda, T. K. Distribution of the pre-solar component in Allende and other carbonaceous chondrites. *Earth Planet. Sci. Lett.* **34**, 209–224 (1977).
